# Sacral stress fracture in an amateur rugby player: a case report

**DOI:** 10.1186/s13256-016-1120-3

**Published:** 2016-11-16

**Authors:** Yasuhiro Takahashi, Takashi Kobayashi, Naohisa Miyakoshi, Eiji Abe, Toshiki Abe, Kazuma Kikuchi, Yoichi Shimada

**Affiliations:** 1 Department of Orthopedic Surgery, Akita Kousei Medical Center, 1-1-1 Iijima-Nishifukuro, Akita, 011-0948 Japan; 2 Department of Orthopedic Surgery, Akita University Graduate School of Medicine, 1-1-1 Hondo, Akita, 010-8543 Japan

**Keywords:** Sacral stress fracture, Athlete, Pediatric, Rugby

## Abstract

**Background:**

Sacral stress fracture is an uncommon cause of back pain. The majority of previously reported cases have been in runners. The purpose of this case report was to describe a case of sacral stress fracture in an amateur rugby player.

**Case presentation:**

A healthy 18-year-old Japanese boy who was a rugby player presented with a 3-week history of lumbago. Sagittal and axial magnetic resonance imaging failed to reveal any reason for lumbago in his lumbar region. On his second presentation, 4 weeks later, his lumbago was so severe that he could not walk without a cane. A second magnetic resonance imaging revealed bone marrow edema with T1-weighted hypointensity and short inversion time inversion recovery hyperintensity at his left sacrum in coronal sections, consistent with stress fracture. Pain was relieved with rest and 1 year later he was able to return to rugby without lumbago or left buttock pain.

**Conclusions:**

Sacral stress fracture can cause low back pain in athletes. Coronal magnetic resonance imaging appears to be an effective option for diagnosis.

## Background

Athletes may experience low back pain. Stress fractures have been described as a cause of back pain, particularly fracture of the pars interarticularis of the vertebrae [1, 2]. Sacral stress fracture is an uncommon cause of back pain, and the majority of previously reported cases have involved runners [3]. The purpose of this case report was to describe a case of sacral stress fracture in an amateur rugby player.

## Case presentation

**Fig. 1:**
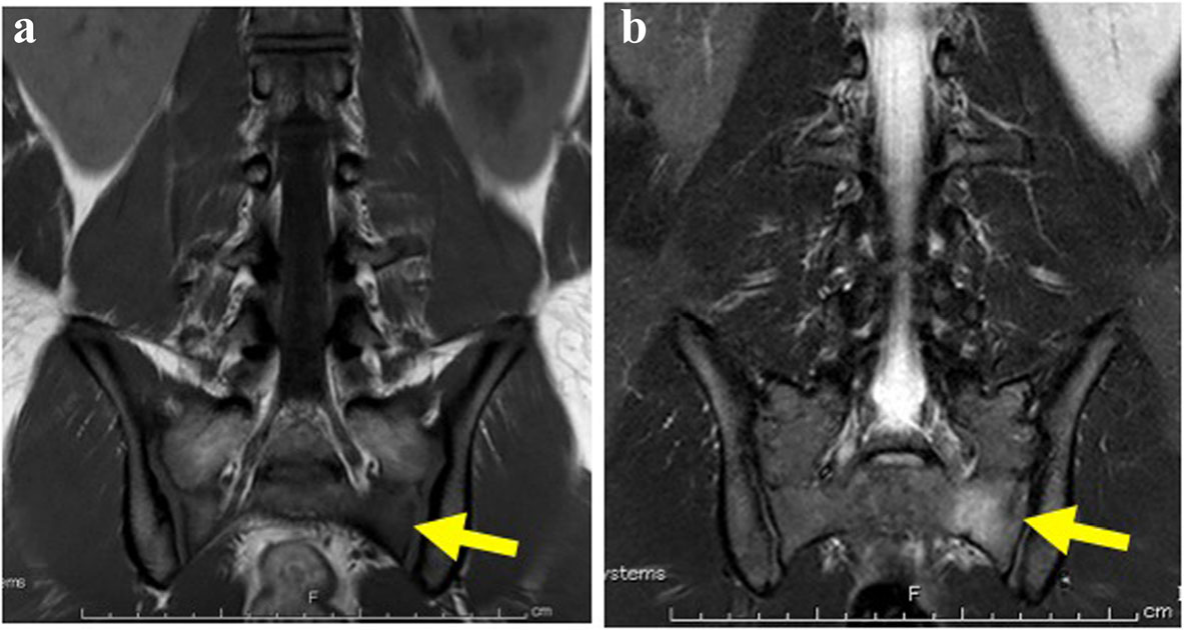
Coronal T1-weighted (**a**) and short inversion time inversion recovery (**b**) images of the pelvis onsecond presentation. Bone marrow edema in the left sacral ala (*arrow*) indicates left sacral fracture

**Fig. 2:**
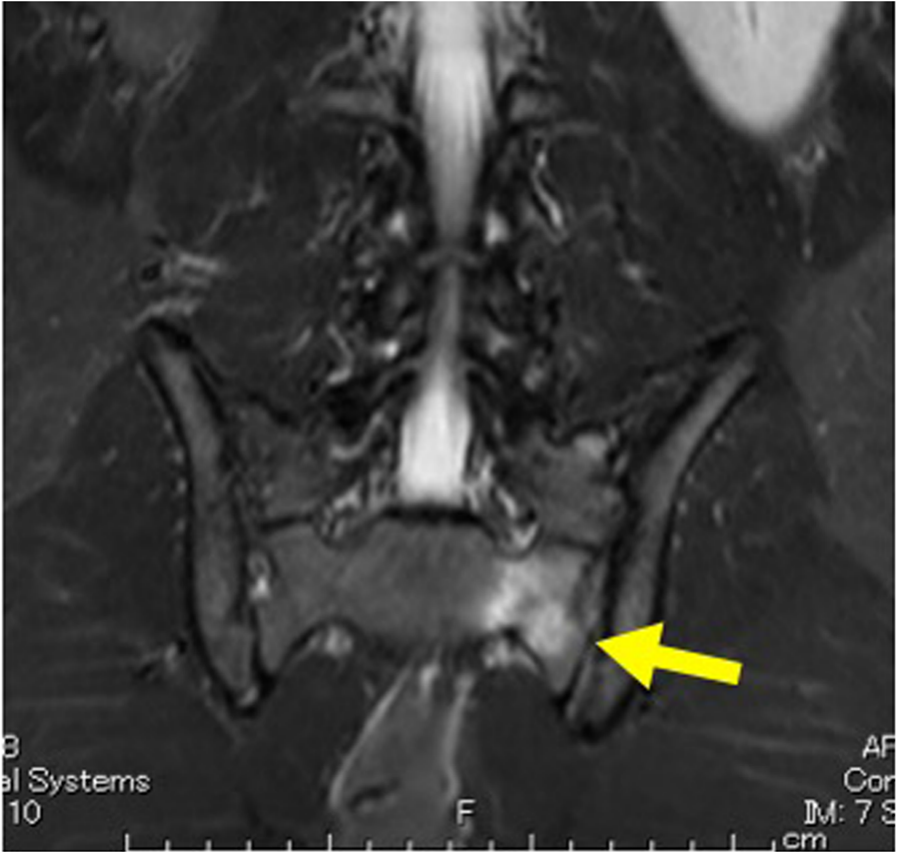
Coronal short inversion time inversion recovery sequence 3 months after the second presentation. Linear area of signal hypointensity in the left sacral ala (*arrow*) indicates the fracture line, with surrounding edema

A healthy 18-year-old Japanese boy who was a rugby player presented with a 3-week history of lumbago. He noticed lumbago after he played rugby. He did not have a clear event of pain onset. Pain had increased over time and was limiting his ability to exercise. Tenderness was identified in his left lumbar paravertebral muscles. Strength, sensation, and reflexes were normal in both his lower limbs. Because previous plain films of his lumbar spine had proven uninformative, magnetic resonance imaging (MRI) of his lumbar spine was performed. Sagittal and axial images failed to show any potential causes of the lumbago in his lumbar region. He continued with his exercise regime and pain increased. He was again admitted to our hospital 1 month after his initial admission. At the time of this second presentation, his lumbago was so severe that he could not walk without a cane. His lumbago was exacerbated on lumbar extension and with load bearing by his left limbs. Tenderness was identified in the region of his left sacroiliac joint. Strength, sensation, and reflexes remained normal in both lower limbs. MRI was again performed, including coronal sections on suspicion of spondylolisthesis. Coronal imaging revealed bone marrow edema with hypointensity on T1-weighted imaging and hyperintensity on short inversion time inversion recovery (STIR) at his left sacrum, consistent with stress fractures (Fig. 1). Pain was relieved with rest, and our patient was able to resume running 4 weeks after second presentation, returning to rugby after a further 2 months. A follow-up MRI at 3 months after his second presentation showed a hypointense area on T1-weighted imaging and a hyperintense area on STIR at his right sacrum in coronal sections (Fig. 2). A follow-up MRI at 6 months after his second presentation showed no evidence of fractures. He was playing rugby without any lumbago or left buttock pain 1 year after his second presentation.

## Discussion

Acute low back pain is common in athletes. Spondylolysis is the most common cause of back pain in adolescents [2]. MRI is the most effective modality for detecting pars injuries [4], and images in the coronal plane are known to be useful in the early diagnosis of spondylolysis [5]. We consider coronal MRI when spondylolysis is suspected. Spondylolysis was not initially suspected in this case, and coronal MRI was therefore not considered. We could not identify any abnormalities on sagittal or axial MRI. On the second admission, lumbar extension was limited and spondylolysis was suspected, so coronal MRI was selected and revealed sacral fractures. We diagnosed stress fracture because of our patient’s gradual onset of lumbago. Coronal MRI offers advantages in not only diagnosing spondylolysis, but also diagnosing sacral stress fractures in athletes.

Sacral stress fractures are an uncommon cause of low back pain. Although the majority of cases previously reported have been in runners [3], and the mean age in the literature is 26 years [3], our patient was an 18-year-old rugby player. Clinical findings are characterized by lumbago, buttock pain, or groin pain [3]. These fractures are thought to often result from training errors when an excessive or rapid increase in the training regimen causes muscle fatigue, with resultant stress on the bone that exceeds its ability to regenerate [6].

Treatment involves rest and activity modification. Most patients are able to return to their normal levels of activity within 4 to 6 weeks [3]. Our patient showed rapid improvement of symptoms with relative rest, and was able to run after 4 weeks and return to rugby after 12 weeks.

## Conclusions

Sacral stress fractures can be a cause of low back pain in athletes. Coronal MRI is an effective option for diagnosing this pathology.

## Abbreviations

MRI: Magnetic resonance imaging
STIR: Short inversion time inversion recovery
